# Hematopoietic stem cell transplantation as rescue therapy for refractory autoimmune retinopathy: a case report

**DOI:** 10.3389/fimmu.2024.1484798

**Published:** 2025-01-07

**Authors:** Wendy Meihua Wong, Yvonne Loh, Hwei Wuen Chan, Warren Fong, Soon-Phaik Chee, Adrian Koh, Graham E. Holder

**Affiliations:** ^1^ Department of Ophthalmology, National University Hospital, National University Health System, Singapore, Singapore; ^2^ Centre for Innovation and Precision Eye Health, Department of Ophthalmology, Yong Loo Lin School of Medicine, National University of Singapore, Singapore, Singapore; ^3^ Curie Oncology and Haematology, Mt Elizabeth Medical Centre, Singapore, Singapore; ^4^ Department of Rheumatology and Immunology, Singapore General Hospital, Singapore, Singapore; ^5^ Eye and Retina Surgeons, Camden Medical Centre, Singapore, Singapore

**Keywords:** antiretinal antibodies, autoimmune retinopathy, electroretinogram, hematopoietic stem cell transplant, plasmapheresis, thymoma, retinal vasculitis

## Abstract

Autoimmune retinopathy (AIR) is a rare, potentially blinding retinal disease that remains a challenging condition to manage when resistant to conventional immune-modulatory approaches. We report clinical and electrophysiological improvement in a 49-year-old patient who underwent an autologous hematopoietic stem cell transplant (aHSCT) for thymoma-associated AIR after experiencing progressive disease despite receiving periocular and systemic steroids, mycophenolate mofetil, baricitinib, tacrolimus, bortezomib, rituximab, plasmapheresis, and intravenous immunoglobulin. The aHSCT had two stages: (i) peripheral blood stem cell harvest following mobilization with cyclophosphamide and granulocyte colony-stimulating factor, and (ii) conditioning regimen with plasmapheresis, rituximab, cyclophosphamide, and anti-thymocyte globulin high-dose therapy, followed by autologous hematopoietic cell infusion of 5.74 million cells. Symptoms of photopsia rapidly abated after undergoing aHSCT, and objective investigations of structure and function similarly demonstrated improvement. At 22 months’ follow-up, she continued to demonstrate the durability of the clinical response. The present report suggests that in judiciously selected patients, HSCT may provide a rescue option for refractory AIR. Further cases are needed to confirm these results.

## Introduction

Autoimmune retinopathies (AIRs) are a group of rare immune-mediated diseases characterized by progressive painless visual deterioration, visual field defects, and electroretinographic abnormalities, in association with circulating anti-retinal antibodies (ARAs) (1, 2). The pathophysiology is postulated to involve the circulating ARAs targeting retinal antigens, causing damage to photoreceptor cells, bipolar cells, or retinal ganglion cells. The two main types are paraneoplastic and non-paraneoplastic AIR; the distinction is important due to the systemic implications (3).

The essential diagnostic criteria for AIR are the presence of serum ARAs and electroretinogram (ERG) abnormalities (4). However, ARAs can be present in other degenerative retinal disorders, systemic autoimmune diseases (ADs), and some healthy individuals, and are not pathognomonic for AIR (5). Whether ARAs initiate the pathogenesis of AIR or represent an epiphenomenon consequent upon retinal dysfunction remains unclear. Electrophysiology is important as ERGs provide objective evidence of the nature, severity, and localization of disease (cone and/or rod photoreceptors, bipolar cells, etc.). In addition, ERG abnormalities may precede the development of structural changes on fundus examination or imaging.

Various immunomodulatory approaches have been employed to alter the disease course in AIR given the presumed immune-mediated nature of the disease. These include local or systemic steroids, antimetabolites (azathioprine and mycophenolate mofetil), T-cell inhibitors (cyclosporin), biologics (rituximab, bortezomib, and IL-6 antagonists), intravenous immunoglobulin (IVIG), and plasmapheresis (6). There is a single case reporting the benefit of nonmyeloablative unmanipulated autologous hematopoietic stem cell transplant (aHSCT), preceded by immune ablation (7). No large-scale randomized controlled trials are available due to the rarity of the condition and the variable natural history. Current available evidence for therapeutic intervention primarily relies on retrospective case series and case reports, and there is no consensus on the most effective treatment regimen (2, 6, 8).

Registry data from the Autoimmune Disease Working Party recorded 3,502 patients undergoing aHSCT, between 1994 and 2021, for various severe ADs (9). The rationale of using aHSCT for AD is to restore immune self-tolerance by eradicating autoreactive immune cells with a conditioning regimen. This is followed by immune reconstitution from myeloid or lymphoid progenitor cells to “reset” the immunological memory (10, 11). There are myeloablative and non-myeloablative conditioning regimens. For AD, the conditioning regimen has a greater emphasis on safety than the regimens designed for malignancies, and the goal of conditioning is immune ablation, not myeloablation. Therefore, a non-myeloablative conditioning regimen with reduced regimen-related toxicity is deemed more suitable for AD (12, 13). In certain ADs, such as relapsing–remitting multiple sclerosis, aHSCT is considered a standard treatment because it achieves sustained remission and neurological improvement with an acceptable safety profile (9, 11, 14). Other major indications for aHSCT are systemic sclerosis and Crohn’s disease, and there is growing evidence for rarer indications such as systemic lupus erythematosus, anti-neutrophil cytoplasmic antibody (ANCA)-associated vasculitis, Takayasu arteritis, and Behcet’s disease (9, 15–17). The present report provides the outcomes of aHSCT in a patient whose central vision was under threat from refractory AIR.

## Case report

### Medical history prior to autologous stem cell transplant

A 49-year-old female patient presented to our institution after receiving treatment at two other healthcare facilities over the previous 18 months. She had a history of thymoma, diagnosed following onset of unilateral ptosis from ocular myasthenia gravis, and had undergone a maximal thymectomy. Histopathological evaluation revealed a stage 1A Masaoka Type B thymoma. A month after surgery, although the ptosis had improved, she developed bilateral photopsia, more prominent in her right eye (RE). At that stage, uncorrected visual acuity was 20/30 bilaterally with normal intraocular pressures and anterior segment biomicroscopy. Posterior segment examination showed bilateral disc hyperemia and peripheral retinal vasculitis, confirmed on fundus fluorescein angiography (FFA). Optical coherence tomography (OCT) showed disruption of the ellipsoid zone in the peripheral macula, and it was worse in her RE.

ARA testing had been positive for antibodies directed towards carbonic anhydrase II, Rab6, aldolase, enolase, HSP60, and TULP1, and a diagnosis of thymoma-associated AIR has been made. There were elevated anti-nuclear antibody titers (>1:640 with a homogeneous pattern) and an inverted CD4/CD8 ratio of 0.61. C-reactive protein, erythrocyte sedimentation rate, extractable nuclear antigen profile, ANCA profile, T-spot TB, TB quantiferon, treponema pallidum particle agglutination assay, and VDRL were normal.

**Figure 1:**
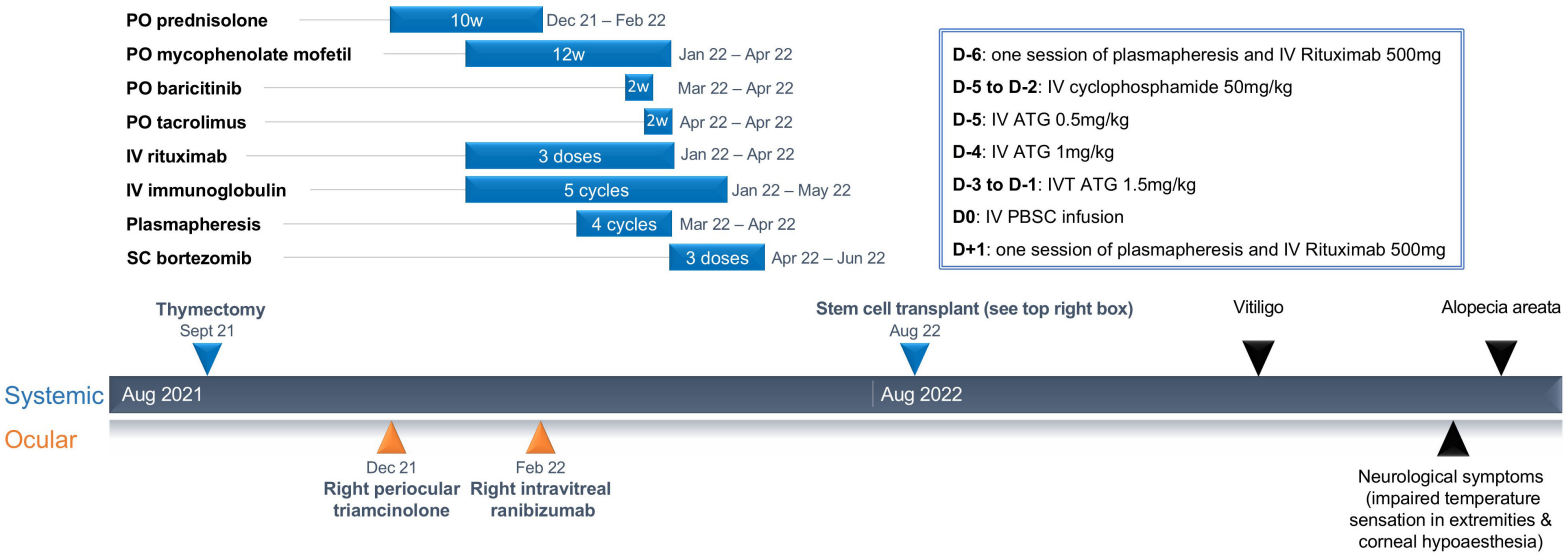
Summary of treatment and duration of treatment leading up to stem cell transplant, and secondary autoimmune diseases after.

The patient had received numerous immune-modulatory treatments prior to presenting to our center (**Figure 1**). She had initially been treated with periocular triamcinolone acetonide and commenced on 50 mg of oral prednisolone (dose of 1 mg/kg) with gradual tapering over 10 weeks. Her disease had continued to be active despite treatment with mycophenolate mofetil, baricitinib, tacrolimus, bortezomib, rituximab, IVIG, and plasmapheresis. FFA, repeated after 4 months of immunosuppression, continued to show similar retinal vascular leakage in both eyes. Antibody testing, repeated at the same time, was positive for several ARAs (30-kDA, 35-kDA, 40-kDA, 44-kDA, 46-kDA, 76-kDA, and 123-kDA proteins) and anti-optic nerve antibodies (40kDA,35kDA,and 123kDA).

**Figure 2:**
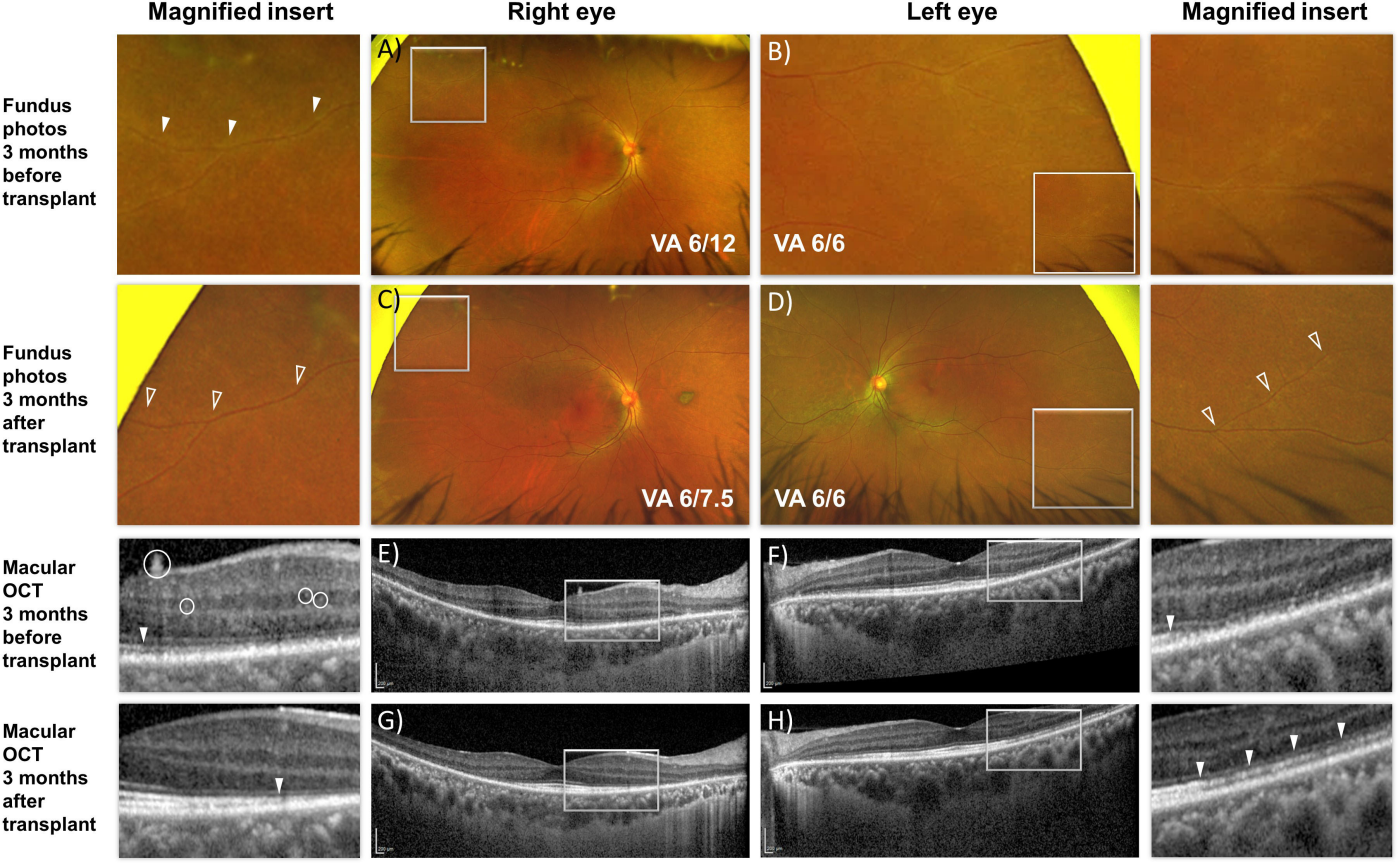
En face fundus images **(A–D)** and cross-sectional optical coherence tomography b-scan **(E–H)** of the macula of both eyes before and after autologous hematopoietic stem cell transplant. **(A, B)** Peripheral vascular sheathing was present in both eyes (magnified insert). **(C, D)** Resolution of the peripheral vascular sheathing 3 months after transplant. **(E, G)** Hyper-reflective dots within the retina and on the retinal surface (white circles) resolved after transplant accompanied by improvement in reflectivity from the ellipsoid zone corresponding to the photoreceptors (solid arrowhead). **(F, H)** Improvement in reflectivity from the ellipsoid zone was similarly present in the left eye 3 months after transplant.

**Figure 3:**
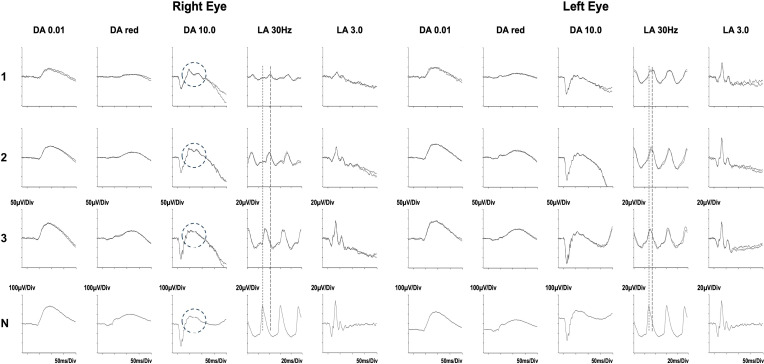
Serial electrophysiological tests performed before (rows 1 and 2) and after the autologous stem cell transplant (row 3). Shown are Standard ERGs as recommended by the International Society for Electrophysiology of Vision. DA = dark adapted; LA = light adapted; the numbers indicate stimulus strength in cd.s/m^2^.The ERGs after 6 months of immunosuppression (24 May 2022, row 1) and repeated 10 days prior to her transplant (12 Aug 2022, row 2) show bilateral generalized retinal cone system dysfunction and severe delay (LA 30 Hz, peak time delay indicated by the interrupted lines). There had been minimal improvement during that time despite ongoing treatment. In addition, in the right eye, the b-wave of the DA 10.0 response showed an abnormal bifid waveform (circled). The LA 30 Hz ERGs 3 months after her transplant (15 November 2022, row 3) show substantial improvement in the delay in the right eye, and normalization in the left eye. There is additional resolution of the abnormal bifid waveform in the DA10.0 of the right eye (circled) to the waveform present in the normal subject (N). Data from a representative normal subject appear for comparison in the lower row (N).

The fundus appearance and OCT imaging on presentation to our center are shown in **Figure 2**. As there continued to be features of disease activity despite 6 months of immunosuppressive treatment, electrophysiological examination was performed at presentation (**Figure 3**, row 1), and repeated 2 months later (**Figure 3**, row 2). There was generalized retinal dysfunction bilaterally, with marked cone system involvement, worse in the RE. Photopic 30-Hz flicker ERG, which reflects generalized retinal cone system function, was markedly delayed in her RE at 42 ms (41 ms on repeat testing) and mildly delayed in her left eye at 33 ms (32 ms on repeat testing).

There was no known family history of vision impairment. The patient was well-informed about her condition and proactively researched her condition, the immune system, and the mechanism of action of her different treatments. She was acutely aware of her ongoing photopsias and that it was approaching her central vision. Additionally, the patient kept a meticulous record of all her test results and tracked the progression evident on structural and functional testing. As her condition continued to deteriorate despite trying multiple lines of immunosuppression, she elected to undergo an aHSCT. The patient was counseled about the risks of aHSCT, and understood the attendant risks including mortality.

## Methods

The patient underwent medical evaluation with an echocardiogram and pulmonary function test before undergoing aHSCT. The aHSCT had two stages: (i) mobilization and peripheral blood stem cell (PBSC) harvest, and (ii) high-dose therapy with autologous hematopoietic cell infusion. The patient received a combination of IV cyclophosphamide 2 g/m^2^ and subcutaneous filgrastim, a recombinant human granulocyte colony-stimulating factor (G-CSF). This regimen is widely used in both cancer and ADs for mobilization of PBSCs (18, 19). A combination regimen was selected because the use of G-CSF alone may trigger flares of AD (20, 21). Cyclophosphamide aids stem cell mobilization by inducing mild cytopenia, which stimulates the marrow to increase the division of stem cells. The PBSCs harvested via apheresis on day 10 after cyclophosphamide had a yield of 5.74 × 10^6^ CD34+ cells/kg body weight and were cryopreserved.

The conditioning regimen was one session of plasmapheresis (on day −6); IV rituximab 500 mg (on day −6 and day +1); IV cyclophosphamide 50 mg/kg (on day −5 to day −2); and IV rabbit anti-thymocyte globulin 0.5 mg/kg (on day −5), 1 mg/kg (on day −4), and 1.5 mg/kg on (day −3 to day −1). This conditioning regimen has been used in aHSCT of patients with neuromyelitis optica (NMO), another antibody-mediated AD that affects the eye (22). Plasmapheresis reduces pre-formed antibodies, while rituximab and anti-thymocyte globulin are postulated to deplete B and T cells (23). IV PBSCs were infused on day 0. She achieved neutrophil engraftment on day +10. Platelets recovered to >20 on day +10 and >50 on day +11. Her transplant course was complicated by neutropenic fever that settled promptly with empirical antibiotics, and she was discharged well by day +11. She also had asymptomatic low-level CMV viremia detected on PCR that resolved without treatment.

## Results

### Clinical evaluation

Shortly after undergoing the transplant, the patient reported rapid resolution of her photopsia. Fundus examination showed resolution of the retinal vasculitis-related vascular sheathing (**Figure 2**).

### Optical coherence tomography

There was restoration of reflectivity in the band corresponding to the ellipsoid zone of the photoreceptors on structural imaging and reduction in the likely inflammation-related intra-retinal hyperreflective foci (**Figure 2**).

### Static automated perimetry

**Figure 4:**
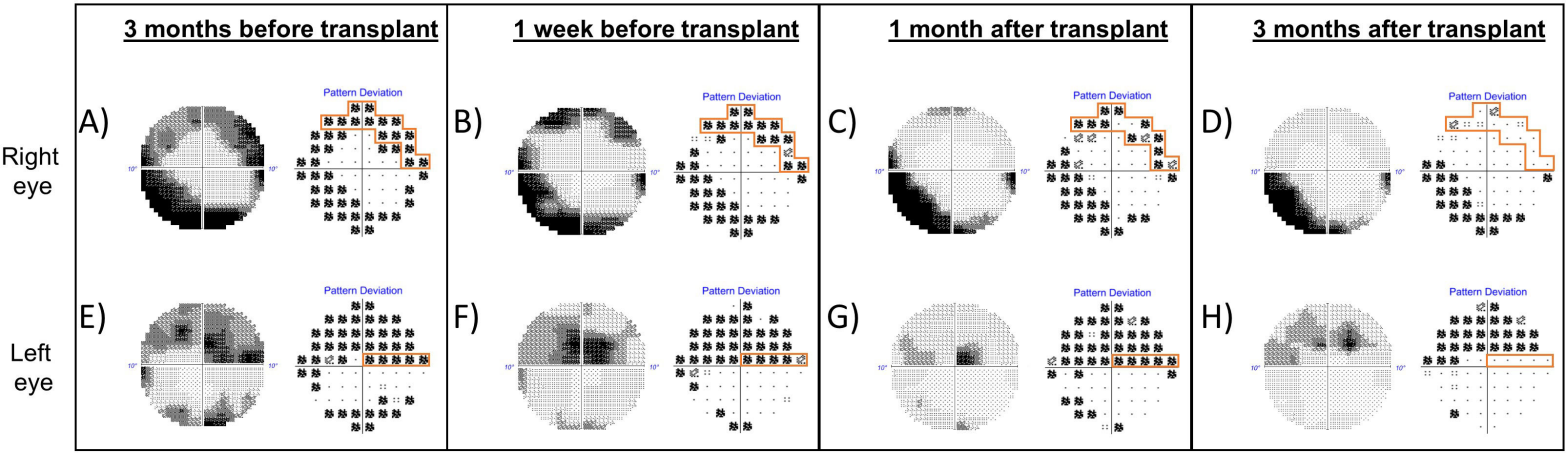
Grayscale map and pattern deviation plot of Humphrey visual fields (10-2 strategy) of the right eye (top row) and left eye (bottom row), before and after autologous stem cell transplant. A reproducible superior scotoma present in the right eye prior to transplant (**A, B**, outlined in orange) demonstrated improvement 1 month after transplant **(C)** and near-complete resolution of the defect 3 months after transplantation **(D)**. **(A–D)** The inferior arcuate defect in the right eye was persistent. There was a reproducible superior visual field defect in the left eye, which threatened fixation before transplant and persisted even 1 month following transplant **(E–G)**. Three months after transplant, there was improvement in the scotoma close to fixation **(H)**.

Visual field testing repeated 3 months after the transplant showed increase in mean deviation scores bilaterally (RE −8.86 dB from −13.91 dB; LE −7.43 dB from −11.51 dB) and reduction in the size of the scotoma in her RE (**Figure 4**).

### Electroretinography

Electroretinography showed profound improvement in RE cone system function and significant improvement in the left eye. There was also rod system improvement with reversion to a normal waveform of the dark-adapted bright flash b-wave (**Figure 3**). See legend for further details.

### Follow-up

Cutaneous manifestations of generalized vitiligo and alopecia areata developed at 6 and 12 months, respectively, after HSCT. Baricitinib was restarted by the dermatologist, but because of incomplete resolution of vitiligo following 4 months of treatment, this was switched to tofacitinib. Improvement in the alopecia was observed.

The patient also experienced neurological symptoms, including impaired temperature sensation in her extremities and bilateral corneal hypoesthesia at 10 and 12 months, respectively, after HSCT, which were managed conservatively.

## Discussion

This report demonstrates a clear response following HSCT in a patient with AIR, in whom a variety of immunomodulatory therapies had been unsuccessful. The criteria for response were objective electroretinographic demonstration of improved retinal function, psychophysical improvement in visual fields, and morphological improvement on OCT imaging. The patient also reported rapid cessation of photopsia. One notable feature in this patient was the resolution of an abnormal electroretinographic b-wave appearance (see **Figure 3**), which has been associated with progressive AIR (Mantel et al., LE, **Figure 4B**) (24).

HSCT is a complex procedure that involves ablation of the host hematopoietic system through chemotherapy and immunotherapy followed by reconstruction of a new system by transplanting hematopoietic stem cells (25). Although its first and most common application is the treatment of onco-hematological disease, HSCT is increasingly being explored as a potential treatment for aggressive ADs (26, 27). While it is challenging to establish that ARAs are the cause of retinal damage in AIR rather than an epiphenomenon, it is suspected that the initiating event was thymic dysfunction, with loss of self-tolerance and resultant production of autoantibodies leading to both myasthenia gravis and AIR (2). A conditioning regimen was therefore selected to target both self-reactive B and T cells. Rituximab and plasmapheresis were utilized to target pre-formed antibodies and B cells, and anti-thymocyte globulin targeted the T cells (28). The safety profile of this regimen has been documented for other immune-related disorders, such as NMO (26). A prospective open-label cohort study of NMO enrolled 13 patients, of whom 12 did not have any active coexisting ADs. In this group, where 11 patients were more than 5 years post-transplant, 80% remained relapse-free off all immunosuppression (22).

A study to evaluate hematopoietic stem cell transplantation in autoimmune-related retinopathy and optic neuropathy (ARRON) syndrome was initiated in 2004 and terminated 8 years later after recruiting two subjects (clinicaltrials.gov identifier NCT00278486). Although no publications were specifically linked, there is a single case report of a patient with ARRON who had been refractory to treatment with steroids, methotrexate, plasma exchange, IVIG, and cyclophosphamide between 2001 and 2003 (7). Improvement was suggested to be present in the ERGs of that case, but the reported changes were minor and the values stated may fall within test–retest variability. Our patient had several notable differences from the previously reported case with a different clinical phenotype, in particular the absence of any clinical or electrophysiological evidence of optic nerve dysfunction, notwithstanding the presence of anti-optic nerve antibodies; the presence of ARAs is not uncommon in the sera of normal humans and the same may apply to anti-optic nerve antibodies (29). There were further differences in disease severity at the time of HCST; our patient had not developed irreversible structural damage leading to central vision loss. Finally, while intravenous cyclophosphamide was used in both cases, the only other immune-ablative drug used in the published case was CAMPATH-1H. CAMPATH-1H is also known as alemtuzumab, and is a humanized monoclonal antibody targeting CD52 on surfaces of lymphocytes, eosinophils, and monocytes, facilitating cell destruction through complement activation and antibody-dependent cellular cytotoxicity (30, 31). The regimen used for the present case, along with its rationale, is detailed above.

Despite access to multiple modern immune-modulating therapeutic agents, the present patient had failed to show significant improvement prior to the HSCT. At the time of writing, the patient has shown durable clinical response for 22 months. Of note, secondary ADs are reported to complicate between 2% and 14% of aHSCTs performed for an AD (28). The postulated mechanisms for a secondary AD to develop include a loss of peripheral tolerance after conditioning (perhaps by deletion of regulatory cells), proliferation of autoreactive cells after HSCT by homeostatic expansion, a failure of negative selection during *de novo* thymic ontogenesis of T lymphocytes, or accumulation of mutations during increased proliferation of lymphocytes (32). During the informed consent process, it is important to have a candid discussion about the potential risks of secondary ADs, as well as the risk of transplant-related mortality, which is estimated to be approximately 1.3% with incorporation of less myeloablative regimens, center experience, and center accreditation (33). Cardiovascular complications can occur acutely, with the most frequent being arrhythmias such as atrial fibrillation and flutter and long-term complications include heart failure; a cardiovascular evaluation prior to aHSCT is thus of paramount importance (34). Patients of child-bearing potential need to also be aware of the risks of temporary or permanent ovarian/testicular failure and infertility following aHSCT (14). In view of the risks of aHSCT, judicious patient selection is important and should be considered where the disease is sight-threatening, standard treatments have failed, and there are no alternative safer therapeutic options. Although randomized controlled trials would be ideal to establish the safety and efficacy of autologous HSCT, it is important to recognize that the inherent challenge is the rarity of IRDs, which is estimated to account for under 1% of all cases even at a tertiary uveitis and ocular immunology clinic (1).

In conclusion, this case report demonstrates that escalation to HSCT in a patient with aggressive AIR that had failed to respond to advanced immunotherapy and threatened central vision was successful in abating the symptoms and led to objective improvement in both structural and functional investigations. Those improvements have been sustained over a 22-month follow-up period. Other symptoms, which may or may not be a direct consequence of the HSCT, have responded to appropriate treatment. Further cases are needed to confirm HSCT as a viable rescue option for refractory AIR.

## Data Availability

The raw data supporting the conclusions of this article will be made available by the authors, without undue reservation.
